# Beyond the Microscope: Integrating Liquid Biopsies into the Molecular Pathology Era of Endometrial Cancer

**DOI:** 10.3390/ijms26167987

**Published:** 2025-08-19

**Authors:** Miguel Perez, Luis Lorenzo Carvajal, Andres Wong, Robert Poppiti, Roberto Ruiz-Cordero, Amilcar A. Castellano-Sánchez, Hisham F. Bahmad

**Affiliations:** 1 Herbert Wertheim College of Medicine, Florida International University, Miami, FL 33199, USA; mpere943@med.fiu.edu (M.P.); llore049@med.fiu.edu (L.L.C.); awong075@med.fiu.edu (A.W.); 2 Arkadi M. Rywlin M.D. Department of Pathology and Laboratory Medicine, Mount Sinai Medical Center, Miami Beach, FL 33140, USA; robert.poppiti@msmc.com; 3 Department of Pathology, Herbert Wertheim College of Medicine, Florida International University, Miami, FL 33199, USA; 4 Department of Pathology and Laboratory Medicine, University of Miami Miller School of Medicine, Miami, FL 33136, USA; rxr1314@med.miami.edu

**Keywords:** endometrial cancer (EC), liquid biopsy, circulating tumor cells (CTCs), circulating tumor DNA (ctDNA), cell-free DNA (cfDNA), molecular, review

## Abstract

Endometrial cancer (EC) is the most common gynecologic malignancy in developed countries, with a growing incidence and significant molecular heterogeneity that challenges traditional diagnostic and management paradigms. While histopathological assessment remains the gold standard for diagnosis, emerging liquid biopsy technologies provide promising non-invasive alternatives for tumor detection, molecular profiling, and disease monitoring. This review comprehensively explores the current landscape and clinical utility of liquid biopsy analytes—including circulating tumor cells (CTCs), circulating tumor DNA (ctDNA), cell-free DNA (cfDNA), extracellular RNAs, and exosomes—in the context of EC. We discuss the evolving role of pathologists in integrating molecular data with histomorphological features to enhance diagnostic precision, prognostic stratification, and therapeutic decision-making. Novel technologies such as methylation-based assays, tumor-informed ctDNA sequencing, and tumor-educated platelets (TEPs) are highlighted for their diagnostic accuracy and potential for early detection. Furthermore, we summarize key clinical trials and future directions aimed at validating liquid biopsy platforms for routine clinical implementation. As EC care transitions toward a precision oncology model, the integration of liquid biopsy with traditional surgical pathology offers a transformative approach to individualized and personalized patient management.

## 1. Introduction

Endometrial cancer (EC) represents the most prevalent gynecologic malignancy in developed nations, with a steadily rising incidence over the past two decades, particularly among postmenopausal women [1,2]. EC incidence continues to rise in the United States (U.S.) and globally, with around 69,000 uterine cancers projected in 2025 in the U.S. alone (the majority endometrial carcinoma) [2,3,4]. Disparities persist: Black women experience higher incidence of aggressive, non-endometrioid histologies and disproportionately higher mortality (Siegel et al., 2025; SEER, 2025) [2,3]. Recent analyses also suggest faster incidence growth among younger women and certain racial/ethnic groups, underscoring a need for accessible, patient-centered diagnostic pathways (Rodriguez et al., 2025) [4]. It originates from the endometrial lining of the uterus and is now recognized as the fourth most common cancer in women worldwide, following breast, lung, and colorectal cancers, and the fifth most common cause of cancer death [2]. Epidemiologic trends show a disproportionate increase in incidence among Black women, with annual growth rates exceeding 2% to 3%, compared to 0.6% in White women [1,5]. Diagnosing EC typically requires invasive procedures such as hysteroscopy, dilation and curettage (D&C), or endometrial biopsy. While these methods generally yield a definitive histopathologic diagnosis, they have their limitations. Procedural risks—including uterine perforation, infection, and complications related to anesthesia—still exist. In addition, due to intratumoral heterogeneity, particularly in cases of mixed histologic subtypes of EC, limited biopsy sampling may fail to capture the full spectrum of the tumor morphology, leading to incomplete or misleading diagnosis. Therefore, there is an unmet need for minimally invasive, cost-effective, and accurate modalities that can detect, monitor, and predict EC progression and therapeutic response.

In several solid tumors, blood-based biomarkers have played a pivotal role in detection, disease monitoring, and therapeutic response assessment. For instance, CA125 in ovarian cancer, carcinoembryonic antigen (CEA) in colorectal cancer, and prostate-specific antigen (PSA) in prostate cancer are routinely used in clinical practice as cost-effective, minimally invasive tools to guide patient management. Although these markers lack absolute sensitivity and specificity, they have demonstrated substantial value in tumor diagnosis and disease monitoring [6,7]. The clinical integration of these biomarkers has contributed to significant improvements in outcomes and is reflected in the progressive decline in mortality rates associated with these cancers. This paradigm underscores the urgent need for similarly reliable circulating biomarkers in EC, where no FDA-approved serum or plasma-based markers currently exist, to enable earlier detection of recurrence, assess therapeutic response, and ultimately improve patient survival.

**Figure 1:**
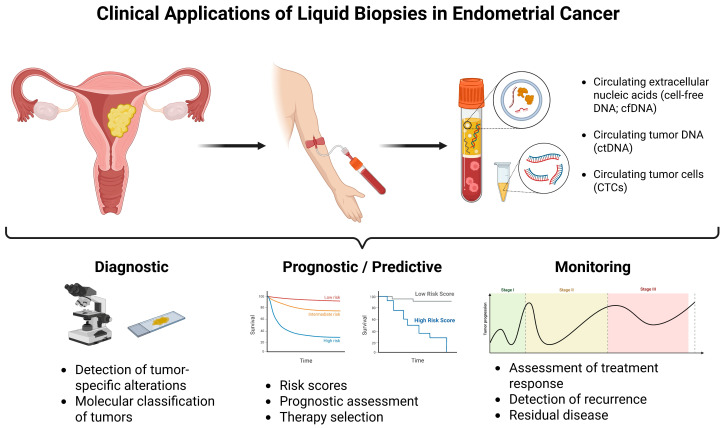
Application of Liquid Biopsy in Endometrial Cancer. This schematic illustrates the potential clinical applications of liquid biopsies in the management of endometrial cancer. Blood-derived biomarkers such as circulating tumor cells (CTCs), circulating tumor DNA (ctDNA), microRNAs, and exosomes can be analyzed using advanced technologies to provide non-invasive insights across three key domains: (1) Diagnostic applications, including detection of tumor-specific alterations and molecular classification of endometrial cancer subtypes; (2) Predictive and prognostic applications, including therapy selection based on molecular profiles and risk stratification; and (3) Monitoring applications, such as assessment of treatment response, detection of recurrence, and identification of residual disease. Integration of liquid biopsy into clinical workflows may enhance personalized treatment strategies and improve patient outcomes. Created in BioRender. Bahmad, H. (2025) https://BioRender.com/ugwjkm5.

Liquid biopsies including the genomic profiling of circulating tumor cells (CTCs), circulating tumor DNA (ctDNA), cell-free DNA (cfDNA), cell-free RNA (cfRNA), microRNA (miRNA), and exosomes, among others offer a real-time, non-invasive approach into studying the tumor’s genetic and epigenetic landscape [8,9]. These techniques have demonstrated the ability to detect actionable mutations, assess disease burden, monitor minimal residual disease (MRD), and predict recurrence well before radiographic or clinical signs emerge [10,11]. Liquid biopsies have the potential to overcome the limitations of tissue sampling by capturing tumor heterogeneity and clonal evolution over time, an essential feature in the management of EC given its molecular diversity [12,13,14,15,16,17,18]. As the field moves toward precision oncology, the integration of liquid biopsy technologies into routine clinical workflows represents a promising frontier for individualized diagnostics, prognostication, and therapeutic monitoring in EC (Figure 1).

## 2. Endometrial Carcinoma in the Molecular Era: Diagnostic Refinements and Emerging Roles for Pathologists

The “World Health Organization (WHO) 2020 Classification of Female Genital Tumors” introduced an integrated morphological and molecular approach to classify ECs, reflecting recent advances in genomic characterization and prognostic stratification [17,19]. Traditionally, ECs were categorized into two broad pathogenetic types, as described by Bokhman in 1983: type I tumors, which represent up to 70% of EC cases, are estrogen-driven, low-grade, and typically exhibit endometrioid histology; and type II tumors, which are estrogen-independent, high-grade, and include serous and clear cell carcinomas [20,21]. However, this binary model had been increasingly viewed as oversimplified due to the significant overlap in clinical behavior, morphology, and molecular alterations between the subtypes [22,23,24].

Endometrioid carcinoma remains the most common subtype, accounting for approximately 75–80% of all EC cases [12]. These tumors often demonstrate glandular differentiation, focal squamous metaplasia, and harbor molecular alterations including *PTEN*, *ARID1A*, *PIK3CA*, and *CTNNB1* genes [17,21]. In contrast, high-grade carcinomas, including serous carcinomas, are typically *TP53*-mutated, genomically unstable and represent an aggressive subtype associated with poor prognosis, even when confined to the endometrium [25]. Clear cell carcinoma, although rare, shares similar aggressive behavior and frequently harbors *TP53*, *PPP2R1A*, and *PIK3CA* mutations [26]. Undifferentiated and dedifferentiated carcinomas are characterized by a patternless, sheet-like growth of discohesive tumor cells with an aggressive clinical course. They harbor somatic mutations in *PIK3CA*, *CTNNB1*, *TP53*, *FBXW7*, and *PPP2R1A* genes and present significant diagnostic challenges due to overlapping histologic and molecular features [27,28]. In addition, loss-of-function mutations affecting key members of the SWI/SNF chromatin remodeling complex—notably *SMARCA4* (*BRG1*), *SMARCB1* (*INI1*), *ARID1A*, and *ARID1B*—are implicated in the process of dedifferentiation. These alterations are observed in approximately two-thirds of dedifferentiated carcinomas and in nearly half of undifferentiated carcinomas, often resulting in complete loss of protein expression within the undifferentiated component [29,30,31,32,33,34]. Carcinosarcoma, previously considered a mixed epithelial–mesenchymal neoplasm, is now recognized as a metaplastic form of EC characterized by transdifferentiation (epithelial-to-mesenchymal transition; EMT) during tumor evolution [35,36,37]. This reclassification is a result of the genomic studies showing shared clonal origin with high-grade carcinomas and frequent *TP53* mutations [17].

The integration of The Cancer Genome Atlas (TCGA) molecular classification has further revolutionized tumor stratification by defining four distinct genomic subgroups: (1) ultramutated *POLE*, includes tumors with inactivating mutations in *POLE* exonuclease and are associated with excellent prognosis; (2) microsatellite instability-high (MSI-H) hypermutated tumors with intermediate prognosis; (3) copy number-low (endometrioid-like) tumors, which correspond to those tumors with low copy-number aberrations, also with intermediate prognosis; and (4) copy number-high (serous-like) tumors, frequently *TP53*-mutated, with poor outcomes [21,38,39]. This classification has now been translated into routine clinical practice using surrogate markers such as immunohistochemistry (IHC) for mismatch repair (MMR) proteins and p53, along with targeted *POLE* mutation testing [22,40,41,42,43]. Furthermore, the WHO 2020 update has introduced novel tumor subtypes, including squamous cell carcinoma, mesonephric-like adenocarcinoma and gastric-type mucinous carcinoma, while also consolidating previously distinct entities—such as mucinous carcinoma—into endometrioid variants based on molecular profiling [17]. Mesonephric and mesonephric-like adenocarcinomas typically show *KRAS* mutations with gain of 1q, and *ARID1A* mutations [44,45,46]. Important advances in the classification of mesenchymal tumors have also emerged, particularly with respect to high-grade endometrial stromal sarcomas and leiomyosarcomas, where integration of gene fusions and next-generation sequencing (NGS) has decreased misclassifications as undifferentiated uterine sarcoma [47,48,49].

These classification refinements highlight the pivotal role of molecular diagnostics in supporting the histomorphologic assessment and enabling accurate tumor stratification, risk assessment, and treatment planning. They also lay the foundation for integrating liquid biopsy approaches that interrogate tumor-derived DNA, RNA, and cells in peripheral blood or other biofluids, offering non-invasive, real-time insights into tumor heterogeneity, progression, and therapeutic response [8,10,50,51,52,53,54,55,56]. However, despite advances in cancer genomics, the molecularly guided management of EC remains less developed compared to other malignancies such as breast and lung cancers, where established molecular biomarkers inform treatment decisions, particularly in the setting of advanced or recurrent disease [57,58,59,60].

The rise of liquid biopsy technologies has expanded the role of pathologists in cancer care, necessitating having some expertise to integrate molecular data with traditional histopathological findings. As clinical management of EC increasingly incorporates molecular signatures alongside traditional tissue evaluation, pathologists play a central role in bridging histopathologic interpretation with data derived from non-invasive molecular assays such as liquid biopsies [61]. A study led by the ctDNA technology work group of the European Liquid Biopsy Society (ELBS) showed that the interpretation and reporting of ctDNA results require specialized expertise when applying liquid biopsies to clinical decision-making [62]. This is due to dedicated complex guidelines regarding the standards for the interpretation of liquid biopsy mutation panels and relaying the results to the oncology team [63]. This integration is essential for accurate diagnosis, prognosis, therapy prediction, and tumor monitoring. The evolution of these technologies demands that pathologists keep up to date with new competencies in digital tools, molecular platforms, and computational analysis including those with artificial intelligence [54].

## 3. Liquid Biopsies: Technologies and Biomarkers

**Figure 2:**
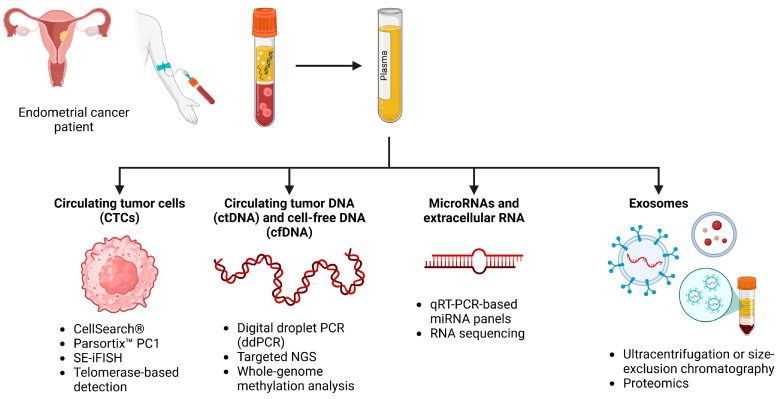
Schematic overview of liquid biopsy strategies in endometrial cancer. Peripheral blood collected from patients is processed to isolate plasma, which contains tumor-derived analytes including circulating tumor cells (CTCs), circulating tumor DNA (ctDNA) and cell-free DNA (cfDNA), extracellular RNAs (such as microRNAs), and exosomes. CTCs are detected using platforms like CellSearch^®^, Parsortix™, SE-iFISH, and telomerase-based detection. ctDNA and cfDNA are analyzed using droplet digital PCR, targeted next-generation sequencing (NGS), and methylation profiling. MicroRNAs and extracellular RNAs are identified through qRT-PCR and RNA sequencing of tumor-educated platelets (TEPs). Created in BioRender. Bahmad, H. (2025) https://BioRender.com/nhvkwe0.

Liquid biopsy refers to the analysis of tumor-derived materials circulating in biofluids such as blood, urine, or uterine aspirates. The key analytes currently investigated in EC include CTCs, ctDNA, cfDNA, extracellular RNAs (e.g., miRNA), and exosomes. Each component offers unique insights into tumor biology, heterogeneity, and clinical progression, while allowing for minimally invasive, real-time disease monitoring. Liquid biopsies in cancer are mainly performed using a peripheral blood sample, which is less invasive, more efficient, and causes less morbidity in the patients compared to conventional tissue biopsies. For the analysis of the biomarkers pertinent to EC, a blood sample would be collected using regular phlebotomy technique. Malignancies that do not arise from the endometrium could potentially require a different type of fluid such as cerebrospinal fluid (CSF) or urine. Once the sample of blood is collected into a specific tube that prevents cell lysis to decrease shedding of nuclear DNA, it is centrifuged at high velocity to separate the whole blood from the plasma, which contains the biomarkers of interest. When needed, an aliquot of plasma can then be frozen until analysis to preserve the integrity of the genetic material. The methods of analysis can differ according to what the target biomarkers are, and what is being captured for analysis, including ctDNA, cfDNA, RNA, exosomes, or CTCs [8]. All these markers reveal useful information about the tumor (Figure 2).

### 3.1. Circulating Tumor Cells (CTCs)

CTCs are viable tumor cells that have detached from the primary tumor and entered the circulation. They provide critical morphological, immunophenotypic, and genomic information, serving as a real-time biopsy for understanding metastatic potential, tumor burden, and disease progression [8,64]. However, their detection is technically challenging due to their low frequency—often as few as one CTC per billion normal blood cells. Several technologies have been developed for CTC enrichment and detection:

- CellSearch^®^, FDA-approved, utilizes immunomagnetic separation (nanoparticles) targeting EpCAM followed by cytokeratin (CK 8, 18, 19; to confirm epithelial origin) and DAPI staining (to confirm intact cells), with CD45 (to exclude leukocytes) [65]. However, it fails to detect CTCs that undergo EMT, which frequently occurs in high-grade EC.
- Parsortix™ PC1 is a microfluidics-based platform that isolates CTCs based on physical characteristics such as size and deformability, independent of epithelial markers [66]. It uses whole blood, and it is usually loaded via a microfluidic cassette into a Parsortix instrument. This method has an advantage over the CellSearch^®^ method due to its lack of dependence on CTCs having to express epithelial markers such as EpCAM. The system is relatively slow, which may make it difficult to work with in the clinical setting, but it would detect CTCs including those that may have lost epithelial markers during EMT.
- SE-iFISH (Subtraction Enrichment and Immunostaining-FISH) combines the removal of leukocytes and erythrocytes with immunostaining and fluorescence in situ hybridization to detect CTCs. It allows for the identification of CTCs regardless of EpCAM expression and provides insights into chromosomal abnormalities. While still under investigation, its ability to detect a diverse range of CTCs makes it a promising tool in EC research [64].
- Telomerase-specific adenovirus-mediated fluorescence detection uses telomerase specific and replication selective adenovirus. In rapidly diving cells such as cancer cells, the activity of telomerase in DNA is highly elevated, which allows us to identify a wide range of tumors without having to do CTCs enrichment, which means concentrating the DNA to a higher density. The virus expresses a green fluorescent protein (GFO) that allows for the direct visualization of the detected tumor cell under a fluorescence microscope [67]. This method is very time-consuming and requires a lot of training for technicians due to its complexity, especially when analyzing larger volumes of body fluids. Lack of standardized clinical validation cutoffs limits its usefulness in clinical practice.

### 3.2. Circulating Tumor DNA (ctDNA) and Cell-Free DNA (cfDNA)

cfDNA is fragmented DNA released into the circulation from normal and apoptotic cells, while ctDNA refers specifically to the tumor-derived fraction of cfDNA. ctDNA carries somatic mutations, methylation patterns, and copy number alterations reflecting the genomic landscape of the tumor, and its levels correlate with disease burden, prognosis, and recurrence [7,9]. Techniques used for ctDNA detection include:

- Droplet digital PCR (ddPCR) allows precise quantification of known mutations or methylated targets in ctDNA with high sensitivity and specificity [68].
- Targeted NGS enables broad mutation profiling and detection of actionable mutations (e.g., *PTEN*, *PIK3CA*, *TP53*, *KRAS*), even in early-stage EC [7].
- Whole-genome methylation analysis is applied to cfDNA to identify epigenetic signatures of endometrial tumors, including early-stage disease, using panels such as ZSCAN12 and GYPC [69,70,71].

Recent studies have demonstrated high concordance between ctDNA mutations and tumor tissue in EC, with ctDNA also serving as an early indicator of recurrence and treatment resistance [9].

### 3.3. Circulating Cell-Free RNA (cfRNA)

Circulating cell-free RNA (cfRNA) sequencing is another emerging technology that enables the detection and characterization of RNA molecules released by tumor cells into the bloodstream. Unlike traditional circulating tumor RNA studies focused on microRNAs or small RNA panels, cfRNA sequencing provides a broader, transcriptome-wide view of tumor-derived RNA signatures, offering potential advantages for early cancer detection, tumor classification, and monitoring treatment response.

Although the application of cfRNA sequencing in EC remains largely unexplored, multiple recent studies have demonstrated its feasibility and utility in various malignancies, setting the stage for its future incorporation into gynecologic oncology. For instance, RARE-seq (Rare And Reproducible Expression sequencing) enabled ultrasensitive cfRNA detection of early-stage lung cancer by amplifying rare transcripts from plasma cfRNA with minimal input volumes [72]. Similarly, SLiPiR-seq introduced a protocol for small-input cfRNA sequencing, enabling pan-cancer detection from as little as 50 µL of plasma [73]. The CCGA (Circulating Cell-free Genome Atlas) project further validated the ability of cfRNA to complement cfDNA in capturing tumor biology, particularly in cancers with low mutational burden, through integrative transcriptomic and epigenomic analysis [74].

Innovative computational approaches have also enhanced the utility of cfRNA. The cfPeak pipeline introduced fragment-level profiling of cfRNA, enabling the prediction of metastatic potential across tumor types [75]. Moreover, LIME-seq highlighted how cfRNA methylation signatures associated with the gut microbiome could distinguish colorectal cancer from controls, suggesting a role for microbiota-derived cfRNA biomarkers [76].

In a separate effort, LOCATE-seq applied long-read nanopore sequencing to plasma cfRNA, revealing novel cancer-associated transcripts and RNA isoforms not captured by short-read sequencing platforms [77]. This technology may be particularly promising for identifying fusion transcripts, alternative splicing events, and non-coding RNA species relevant to tumor biology.

While no studies have yet applied these advanced cfRNA sequencing strategies specifically to EC, the success demonstrated across multiple tumor types reflects the untapped potential of cfRNA in this field. The integration of cfRNA sequencing into EC research could reveal transcriptomic biomarkers for early detection, prognostication, and therapeutic stratification—especially in tumors that may evade detection by DNA-based assays due to low mutation burden or high intratumoral heterogeneity. Future directions should include pilot studies investigating cfRNA profiles in EC patients using NGS platforms and integrating multi-omics approaches combining cfDNA, cfRNA, and proteomic data to improve diagnostic accuracy.

### 3.4. Extracellular RNA and miRNAs

miRNAs are small non-coding RNAs involved in post-transcriptional gene regulation and are frequently dysregulated in cancer [78]. Tumor-derived miRNAs can be detected in serum, plasma, or uterine aspirates and may serve as diagnostic or prognostic biomarkers [79]. Several EC-associated miRNAs, such as miR-200 family and miR-21, have been linked to tumor stage, grade, and lymphovascular invasion [8]. Emerging analytical methods include:

- qRT-PCR-based miRNA panels from plasma or uterine fluid.
- RNA sequencing of tumor-educated platelets (TEPs): A novel approach where platelets modulate their RNA content upon interaction with tumor cells and show promise in detecting EC with more than 95% accuracy [54].

### 3.5. Exosomes

Exosomes are nanoscale extracellular vesicles (30–150 nm) secreted by tumor and stromal cells, containing nucleic acids (DNA, mRNA, miRNA), proteins, and lipids. They facilitate intercellular communication and modulate the tumor microenvironment [80,81]. Exosomal content reflects the originating tumor’s genotype and phenotype and can be harnessed for diagnostic and prognostic purposes. Recent findings indicate that exosomal miRNAs and proteins isolated from plasma or uterine aspirates may distinguish EC from benign conditions and other gynecologic malignancies with high sensitivity [82,83]. Methods include:

- Ultracentrifugation or size-exclusion chromatography for exosome isolation.
- Proteomics (e.g., Liquid Chromatography–Parallel Reaction Monitoring [LC-PRM]) for profiling exosome-derived protein biomarkers such as MMP9 and PKM.

## 4. Mutation Panels and Methylation Profiling

One major focus of liquid biopsies in EC is their ability to detect specific mutations in the ctDNA. Studies have found that alterations in *PTEN*, *PIK3CA*, *KRAS*, and *CTTNB1* are among the most common mutations in EC [21,27,39,50]. A study showed that up to 94% of patients will have at least one of these oncomutations detected by NGS panel [7]. However, only one-third of the mutations detected via liquid biopsy were the same as the mutation present in the tumor. A more recent study done on 21 Slovak EC patients detected oncomutations in the ctDNA of every single patient and were able to match two-thirds of them to the mutations in the tumor [10]. Although mutations including *PTEN*, *PIK3CA*, and *TP53* were identified, the researchers noted more frequent mutations associated with clonal hematopoiesis, specifically *DNMT3A* [10]. However, the clonal hematopoiesis mutations were not present in the matched tumors. Additionally, other studies showed that cfDNA sequencing can also capture valuable information about disease burden and recurrence risk [11,50,84,85,86,87,88]. In this cohort of 44 newly diagnosed EC patients, a detection of baseline ctDNA using a highly sensitive high-depth liquid biopsy sequencing assay almost always matched a patient with an advanced stage of EC and poor progression-free survival [9].

Apart from identifying mutations in ctDNA via NGS, there are other methods to molecularly profile EC detection. Studies have shown how DNA methylation markers can be used to detect EC through collection of urine, cervicovaginal samples, and cervical scrapes [69,71]. By testing with a panel of nine gene promoters using quantitative methylation-specific PCR, one study demonstrated that each marker’s methylation level was significantly higher in EC patients [69]. This method was also able to detect early-stage cancers with sensitivity and specificity around 90%. Furthermore, more accurate methylation tests have been developed recently, targeting other genes like *ZSCAN12*, *GYPC*, *RASSF1A*, and *HOXA9* [70]. Simultaneously, other researchers have looked at a step beyond diagnosis and prognosis and have looked at real-time monitoring of tumor dynamics. ctDNA levels or a recurrence of mutations after treatment can signal a relapse of the cancer before showing up on imaging or symptoms occurring, while clearance of ctDNA signals a positive trend towards recovery/remission [9]. In fact, a sequence or series of liquid biopsies can be used to track the emergence or the resistance to treatment of certain mutations in EC patients [54]. As the field evolves, it is essential for pathologists to interpret molecular profiling, as discrepancies between the liquid biopsy and the morphology of the tumor are still evident.

To provide a comprehensive overview of the emerging clinical relevance of liquid biopsy approaches in EC, we compiled current evidence on circulating biomarkers from primary studies published between 2010 and 2025 (Table 1). This table summarizes key findings from studies evaluating the diagnostic, prognostic, and monitoring utility of ctDNA, cfDNA, CTCs, and exosomal microRNAs (miRNAs). These studies demonstrate the increasing feasibility and translational potential of liquid biopsy in early detection, molecular profiling, and longitudinal monitoring of EC. Notably, several tumor-informed ctDNA assays and methylation-based platforms have shown high sensitivity and specificity, supporting their incorporation into future clinical workflows. However, the heterogeneity in methodology and patient cohorts highlights the need for standardization and validation in larger, prospective trials.

## 5. Clinical Applications of Liquid Biopsy in Endometrial Cancer

### 5.1. Potential Predictive Biomarkers Detectable via Liquid Biopsy

In order to successfully screen, diagnose, and methodically implement treatment approaches for EC based on liquid biopsy data, the identification of relevant biomarkers found through liquid biopsies becomes of utmost importance. One of the markers routinely studied for the screening and monitoring of the disease is human epididymis protein 4 (HE4). It has been documented that HE4 is elevated in most ECs. Data from a comprehensive meta-analysis demonstrates that HE4 possesses high specificity (91%), moderate accuracy (AUC 0.84), low sensitivity (65%), and a high diagnostic Odds Ratio (19.46) at identifying and differentiating cases of EC [97]. These values are in concordance with the findings from another study’s data, which closely corroborates the sensitivity and specificity values of HE4 (67% and 95%, respectively) [98]. These data indicate that HE4 could potentially be a good marker for differentiating EC cases from non-EC cases but is not very useful as a standalone marker for diagnosis.

Another relevant factor when determining a useful biomarker is the correlation between the expression of the marker and the relevant severity of the disease. Ideally, a marker which levels increase with the severity of disease, or is positive in more aggressive disease types could hold promise at preemptively treating and monitoring EC. For example, expression of thyroid transcription factor-1 (TTF-1) in EC CTCs was associated with increased incidences of high grade tumors, increased lymph node and vascular infiltration, and metastasis when compared with CTCs negative for TTF-1 expression [99]. High serum HE4 was also significantly associated with a subset of Type II highly aggressive, poorly differentiated ECs, with decreased overall survival, disease-free survival, and progression-free survival times [98].

An important consideration that pathologists are currently implementing, and will be even more important in the future, is the correlation between quantity of CTCs/ctDNA present in blood samples and predicting tumor aggressiveness as well as patient prognosis. Studying and using this relationship for disease prediction remains one of the hallmarks of the potential in liquid biopsies. Some studies have investigated this relationship, with indication that CTC presence could be useful in predicting tumor behavior in EC. In a recent study, 40 patients with pre-operative EC diagnosis had their peripheral CTC count evaluated. The patients were assigned into either a high-risk group (grade 3, stage pT3–pT4 EC) or an intermediate risk group (grade 2–3, stage pT1–pT2 EC) [100]. It was found that a positive CTC count (15% of patients) on either group was significantly correlated to higher degree of cervical invasion with no significant difference between high-risk and intermediate risk subgroups. Another study also showed that 60% of patients with EC had a positive CTC count, and CTC presence was associated with tumor size greater than 5 cm, worse disease stage, and worse survival expectancy [94]. Other studies correlated between higher CTC counts and degree of deep myometrial invasion and lymph node positivity [84].

### 5.2. Application of Liquid Biopsies to Cervical Cytology Samples for Endometrial Cancer Detection

In recent years, cervical cytology-based sampling, including Papanicolaou (Pap) smears and self-collected cervicovaginal samples, has emerged as a promising platform for liquid biopsy applications beyond the detection of cervical neoplasia. The molecular profiling of supernatants from cervical cytology specimens, particularly cfDNA and cfRNA analysis, has shown great potential in detecting endometrial and even ovarian cancers [101]. These approaches leverage the widespread infrastructure of cervical cancer screening programs, offering a minimally invasive and highly accessible avenue for early cancer detection in gynecologic malignancies.

A study by Wang et al. (2025) demonstrated the superiority of Pap smear-derived cfDNA over plasma-derived cfDNA for detecting EC. Tumor-specific mutations were identified in 77.4% of EC or endometrial intraepithelial neoplasia (EIN) cases using cervical cytology supernatant cfDNA, compared to only 42.9% in matched plasma samples [102].

One of the most notable advances in this domain is the PapSEEK assay, which detects tumor DNA mutations from Pap brush or Tao brush-collected cervical samples. In a landmark study, PapSEEK achieved an overall mutation detection rate of 81% in EC patients, including 78% in early-stage disease. When using the Tao brush, which samples closer to the uterine cavity, the detection rate improved to 93% [103].

Further evidence was presented at the 2025 American Society of Clinical Oncology (ASCO) annual meeting, comparing cfDNA from Pap smear-type cervical samples to matched plasma samples in 16 patients undergoing surgery for EC (including both sporadic and Lynch syndrome-associated cases). Findings revealed that Pap-based samples yielded approximately 5-fold higher cfDNA concentrations than plasma. Importantly, tumor-specific mutations were detected in more than 90% of cases using cervical cytology samples—including around 81% detection in early-stage disease—compared to only 25% in plasma, reaffirming the superior sensitivity of cervical sample-based liquid biopsy [104]. Cervicovaginal methylation tests (e.g., WID-qEC, ZSCAN12/GYPC) have also shown high accuracy in hospital-based symptomatic cohorts (AUC around 0.99; sensitivity 100%; specificity 82.5%; n = 330) [71]. While encouraging for triage of abnormal-bleeding presentations, specificity of 82.5% would reduce positive predictive value in population screening; rigorous multicenter, prospective validation in primary care/community settings is required before screening adoption [69,71].

These studies emphasize the clinical potential of incorporating molecular analysis of cervical cytology specimens into screening strategies for EC. Given the rising interest in self-collection approaches and the infrastructure already established for cervical cancer screening, this strategy holds promise for non-invasive, scalable, and sensitive early detection of EC—particularly in resource-limited settings or populations with limited access to endometrial sampling.

### 5.3. Liquid Biopsies in Clinical Trials

Most clinical trials mainly focus on the role of ctDNA as a predictor of disease aggressiveness and recurrence. Specifically, the methylation of certain genes in EC seems to be the direction in which future research is headed into. The determination of prognostic and clinical validity using cfDNA, ctDNA, and liquid biopsies overall, has been mentioned previously as one of the main factors to be further researched in order to incorporate liquid biopsies into the standard diagnostic and prognostic regiments. The current trials and their specifications can be found in Table 2.

## 6. Challenges, Innovations, and Future Directions

### 6.1. Integrating Liquid Biopsies with Traditional Surgical Pathology

The use of liquid biopsies can be promising to determine the prognosis of the cancer after intervention based on the levels of cfDNA and ctDNA. While histopathological findings can be essential to the diagnosis of cancer, it alone is not able to anticipate disease relapses or treatment resistance [88]. It appears that it would be most useful to integrate both techniques as they can both aid in predicting the best outcomes according to the type of treatment used. If the patient is recommended for surgery, then it would be most useful to know what the chances of a recurrence and the levels of cfDNA are. Studies have proven that ctDNA has a positive and statistically significant correlation with early post-surgery relapses before any clinical signs are visible [88].

In many cancers including EC, mutations in different parts of the tumor can vary throughout. There can be different mutation profiles, some which can be low-level mutations and hence quite difficult to determine relying solely on tissue biopsies. Consequently, when there is molecular heterogeneity and variability of mutations within the same tumor, it is best to use a complementary approach requiring liquid biopsies. A single block of tumor may miss other mutations elsewhere, and this is when liquid biopsies can be used for a more comprehensive molecular profiling [7]. Therefore, it is highly recommended that liquid biopsies be integrated with histopathologic assessment and molecular genetic pathology to determine the best approach in the management of EC.

### 6.2. Overcoming Current Limitations

One of the main setbacks of using the CTC approach is the scant availability and wide variability of CTC detection in the patients’ peripheral blood samples. One way that this is getting addressed is by acquiring the sample from blood supplies closer to the site of tumor residence. One study showed promise in capturing higher number of CTCs by taking blood from the ovarian veins from patients with EC [51]. Meanwhile, peripheral blood samples were also taken, and no CTCs were detected in any of the samples. This indicates a highly efficient method of detecting CTCs in patients whose disease is still not advanced enough to spread peripherally.

Another way of working around the low CTC numbers is using ctDNA to screen and monitor more aggressive and less common EC types like uterine serous carcinoma and carcinosarcoma. Continuously monitoring ctDNA levels for these cancer types allowed for monitoring of disease during and after therapy, prediction of recurrent disease, as well as differentiation between malignant vs. benign processes found at CT follow-up [87]. Using ctDNA might be a good alternative in identifying and monitoring these uncommon types of cancer that might not show up on peripheral CTC blood counts. These findings are corroborated by other researchers, indicating ctDNA could also be used to determine if there is residual tumor mass after resection or treatment, and can also serve as an indicator of recurrence in future disease, serving as a much better predictor of recurrence than conventional standards. They also found that lower ctDNA levels after resection were correlated with worse overall survival as well as cancer stage and grade [87].

### 6.3. The “Needle in a Haystack” Problem and the Challenge of False Negatives

While the clinical utility of ctDNA and cfDNA in EC is increasingly recognized, it is important to acknowledge inherent biological and technical limitations that constrain their broader application, particularly in early-stage disease. One critical challenge is the low abundance of tumor-derived DNA fragments within a vast background of non-tumor cfDNA, often described as a “needle in a haystack” problem [106,107]. In early or low-volume disease, the proportion of ctDNA relative to total cfDNA is extremely low, sometimes falling below the limits of detection of even the most sensitive assays such as ddPCR or ultra-deep NGS. Consequently, a negative ctDNA result should be interpreted with caution, as it does not reliably rule out residual disease or recurrence, especially in the adjuvant or surveillance setting [108]. In such scenarios, the absence of detectable ctDNA may reflect assay limitations rather than true disease absence, resulting in false-negative interpretations.

Furthermore, a significant but underrecognized confounder in ctDNA analysis is clonal hematopoiesis of indeterminate potential (CHIP), a phenomenon in which somatic mutations accumulate in hematopoietic stem cells with age, leading to clonal expansion of leukocyte populations. These CHIP-related mutations, particularly in genes such as *DNMT3A*, *TET2*, and *ASXL1*, can be inadvertently detected in plasma cfDNA and misattributed to the tumor, resulting in false-positive findings and erroneous clinical interpretations [109,110]. This issue becomes particularly problematic in older patients, where CHIP prevalence exceeds 10–20% depending on age and sequencing depth.

To overcome this limitation, the recommended best practice is to perform matched sequencing of leukocyte-derived DNA alongside plasma cfDNA to differentiate CHIP-associated variants from true tumor mutations. This approach—often referred to as “white blood cell correction”—helps distinguish between hematopoietic and tumor-derived signals, improving the specificity of liquid biopsy interpretations [110,111]. However, this dual-sample strategy increases cost, complexity, and turnaround time, limiting its current use in routine clinical practice. As liquid biopsy technologies evolve, incorporating CHIP-aware bioinformatic filters and routine leukocyte profiling will be essential to ensure diagnostic accuracy [112].

### 6.4. Equity, Histologic Subgroups, and Generalizability

EC incidence and mortality show persistent disparities by race/ethnicity, with Black women bearing a disproportionate burden and higher rates of aggressive histologies [2,3]. Self-sampling approaches (urine/vaginal/cervicovaginal) are acceptable and feasible in gynecologic contexts and may reduce access barriers; however, EC-specific, community-based validation remains limited [69,113,114]. ctDNA abundance and detection rates can vary by histology and stage; higher detection has been observed in high-grade serous/carcinosarcoma and advanced disease, with sparser data in stage I endometrioid tumors [87,115]. Stratified reporting is essential to avoid spectrum bias. Current literature is constrained by small n, single-center selection, pre-analytical variability, heterogeneous endpoints, limited external validation, and lack of trials demonstrating change in management or improved outcomes attributable to liquid biopsy. Consensus guidance emphasizes pre-analytical standardization, use of matched WBC (“buffy coat”) for CHIP correction when feasible, and transparent validation metrics [116].

### 6.5. Innovations in Liquid Biopsy Technologies

Future endeavors for researchers should focus on finding better and more accurate methods to use in complementation with liquid biopsies. These efforts will hopefully increase the sensitivity of liquid biopsies at detecting EC and beyond. For example, a group of researchers used ctDNA and Tumor Educated Platelets (TEPs), which are platelets whose transcriptomic profile changes as they interact with tumors, as a screening method for EC [54]. They found that using TEPs yielded an accuracy of 99.7% at differentiating between EC and benign or non-EC cases, whereas ctDNA had an accuracy of 67.5% at differentiating these cases. In another attempt at finding highly sensitive and specific markers through different techniques, another group of researchers used uterine aspirates instead of the more widely used peripheral blood sample to determine relevant markers that could be used in the screening and differentiation of EC vs. non-EC cases [117]. Using LC-PRM, a specific liquid chromatography used for proteomic analysis, the researchers found that the co-expression of proteins metalloproteinase-9 (MMP9) and Pyruvate Kinase (KPYM) yielded a sensitivity and specificity of 94.2% and 87.2% at differentiating EC from non-EC samples. When compared with the histological evaluation of the cells, the data from the proteomic expression was able to be used to correctly diagnose 100% of the patients. They also found that the co-expression of proteins CTNB1, XPO2, and CPG was useful in the discrimination of endometrioid EC from serous EC with a sensitivity of 95% and a specificity of 96% [117].

Other researchers have also expanded on ways to make the processing of the liquid biopsy samples more efficient. For example, droplet digital PCR (ddPCR) was used to maximize the detection of methylated ctDNA sequences in patients with EC [68]. Using this new technique, the researchers were able to find 2 hypermethylated genes (*OXT* and *ZSCAN12*) that could be used as a possible screening test for EC. These sequences were measured to have a sensitivity of 98% and a specificity of 97% at differentiating EC positive samples from non-EC samples. Post-surgical presence of ctDNA using this technique was also correlated with relapse of disease, as well as with pre-surgical FIGO staging of the tumor [68].

### 6.6. Artificial Intelligence

Artificial intelligence (AI) has proven to be useful in many different disciplines and topics, and employing AI in endometrial cancer research has not fallen behind. As mentioned before, a previous study using TEPs was also aided by the use of AI [54]. Researchers used predictive models to determine early detection of EC by employing whole-genome sequencing on fragmentomic samples collected via liquid biopsy of 120 EC patients and 120 controls, to build a four-way algorithm which could predict endometrial cancer [89]. This algorithm was then tested out in 62 patients with EC and 62 controls, to see how good of a predictive value the algorithm had. It was found that the algorithm reported an AUC of 0.96, and 75.8% sensitivity at 96.8% specificity at detecting EC cases from controls. Similar values for sensitivity were found for FIGO stages I-IV (74.4%, 85.7%, 75% and 75%, respectively) [89].

### 6.7. Personalized Oncology and Molecular Tailoring

It should come as no surprise that the usage of liquid biopsies comes with great potential to individualize and personalize treatment plans for patients. Knowing the specific tumor composition either via CTC or ctDNA analysis could give physicians and researchers the exact mutations present in the solid tumor, allowing for correct identification of more or less aggressive tumor types, which may result in different treatment plans. A study demonstrated that using personalized ctDNA probe sequences for patients with excised endometrial and ovarian tumors was highly specific (99%) and moderately sensitive (81%) at detecting the presence of tumor at time of surgery. This approach was also more specific than the current standard of measuring CA125 in serum [11]. However, there was no statistical difference between the accuracy of both tests. The ctDNA probes also allowed for prediction of cancer recurrence 7 months earlier than standard CT imaging. Lastly, the presence of ctDNA after resection and adjuvant therapy was correlated with significantly worse overall survival time and disease-free progression time compared to no ctDNA presence. Using a similar methodology for an individualized panel, another researcher corroborated the aforementioned results in terms of increased disease-free survival and increased tumor relapse in ctDNA-positive patients [90].

These results shine a light on what personalized liquid biopsies could look like for the future of cancer treatment and prognosis prediction. A comprehensive panel that can indicate specific mutations on a patient-by-patient basis in a cost-effective and timely manner can uphold the promise of using individualized cancer monitoring and treatment methods. Being able to reliably identify a recurrent cancer phenotype, a cancer resistant to therapy, or a more aggressive cancer faster than current practices holds promise to modify or specialize different types of treatments depending on the individual. As the future of medicine moves into a more personalized view of patient care, it is important to analyze and carefully evaluate these cutting-edge options for more efficacious treatment.

## 7. Conclusions

The current trend in modern medicine toward less-invasive, cost-effective and more practical methods for cancer detection and surveillance makes liquid biopsies an attractive option for EC [61]. Expanding beyond early cancer detection, liquid biopsies have been shown to be useful in the diagnosis, prognosis, and monitoring of the disease. Nevertheless, it is still an ongoing research subject without a particular gold standard. Starting from sample collection, liquid biopsies for endometrial cancer have been obtained from blood samples, plasma, urine, and even cervicovaginal swabs. Then, different techniques have been employed depending on the molecular target used to detect EC, whether it is ctDNA, TEPs, methylated DNA, or exosomal RNA. These can be analyzed through different methods including NGS, ddPCR, quantitative methylation-specific PCR, and trained machine learning algorithms, among others. However, pathologists must remain at the core of this progress as molecular profiling is as complex as ever. For instance, all the different markers discovered must be used in the right patient at the pathologist’s discretion. Every patient is going to show different markers, and different markers are going to guide the pathologist towards a specific risk assessment.

Thus, the introduction of liquid biopsies into frequent use still needs a tumor’s histomorphological evaluation at its core. Pathologists must remain aware of the possible discrepancies between liquid biopsy findings and the actual tumor characteristics. Therefore, genomic literacy and clinical context must be used in combination when dealing with EC. By merging both together, pathologists remain pivotal not only for the diagnosis of the disease, but also to help guide therapeutic decisions and interpret disease monitoring. Looking forward, the future of pathology lies in embracing and applying newer technologies, while understanding what methods to use and what biomarkers to target to either diagnose or help treat the patient.

At present, liquid biopsy should be positioned as an adjunct in observational practice. Tumor-informed ctDNA can complement conventional imaging to track treatment response and to flag molecular relapses earlier in selected high-risk endometrial cancers while recognizing that current evidence is non-interventional. Cervicovaginal methylation assays such as WID-qEC show promise for triaging symptomatic presentations in hospital settings, but they are not yet validated for general-population screening.

To translate these tools into routine care, the field needs coordinated pre-analytical harmonization (including tube type, time-to-spin, sample volume, and freeze–thaw limits), externally validated multicenter studies that stratify performance by histology, stage, menopausal status, and race/ethnicity, and prospective trials demonstrating that liquid-biopsy-guided decisions change management, such as earlier initiation of adjuvant therapy or justified imaging de-escalation/escalation, with hard clinical endpoints including time-to-treatment change, disease-free survival, and overall survival. Routine mitigation of clonal hematopoiesis of indeterminate potential through matched white blood cell sequencing or validated computational approaches is also essential to minimize false positives.

Nowadays, artificial intelligence has also been implemented to increase efficiency and diagnostic accuracy. Nevertheless, there have been notable limitations that should be taken into consideration when using AI, such as the lack of differentiation between a benign tumor and a malignant tumor. Thus, this calls for a path to action where pathologists remain at the forefront of innovation, as refining liquid biopsies must be carefully confirmed with histopathological expertise.

## Figures and Tables

**Table 1:** Summary of liquid biopsy biomarkers evaluated in endometrial cancer.

Biomarker	Clinical Significance	Type of Sample	Cohort	Technology	References
ctDNA (*PTEN*, *PIK3R1*, *KMT2C*, etc.)	ctDNA mutations detected in 93% of EC or AEH patients; mutations correlated with higher grade and myometrial invasion; 65% concordance with tissue biopsy	Plasma	n = 63	NGS-based ctDNA panel	Esposito et al. (2025) [50]
cfDNA/ctDNA monitoring	High levels of cfDNA and detectable ctDNA predict poor DFS and DSS; longitudinal monitoring identifies early recurrence	Plasma, urine aspirates	n = 198	Targeted NGS (Oncomine™), ddPCR	Casas-Arozamena et al. (2024) [88]
Fragmentomics-based cfDNA	Early detection, clinicopathological subtyping (stage, grade, histology, MSI), and recurrence prediction in EC	Plasma	Training: 120 EC + 120 healthy; Testing: 62 EC + 62 healthy	Low-pass whole genome sequencing + machine learning (ensemble model)	Rao et al. (2024) [89]
ctDNA mutations (including *DNMT3A*, *TP53*, *FGFR2*)	ctDNA mutations were detected in 100% of patients; *DNMT3A* mutations were most frequent. Demonstrated feasibility of liquid biopsy for EC molecular profiling	Plasma	n = 21	Targeted NGS	Kodada et al. (2023) [10]
CTCs	Detectable in ovarian vein during early-stage EC surgery; potential for recurrence risk stratification	Peripheral and ovarian vein blood	n = 10	CellSearch^®^	Francini et al. (2023) [51]
Exosomal miRNAs (hsa-miR-17-3p, hsa-miR-99b-3p, hsa-miR-193a-5p, hsa-miR-320d)	Prognostic exosomal miRNAs predictive of poor survival in EC; identified as potential therapy targets	Plasma (Exosomal miRNA data from TCGA)	n = 566	miRNA-seq + bioinformatic analysis (TCGA/NCBI + KM survival modeling)	Yao et al. (2023) [82]
DNA methylation (e.g., *GYPC*, *ZSCAN12*)	High sensitivity and specificity in detecting EC via self-sampling	Urine, self-sample, cervical scrape	n = 103 EC and 317 controls	qMSP	Wever et al. (2023) [69]
Methylated DNA (WID-qEC: *ZSCAN12*, *GYPC*)	AUC 0.99, sensitivity 100%, specificity 82.5%; validated in hospital cohort	Cervicovaginal self-sample	n = 330	qPCR	Schreiberhuber et al. (2023) [71]
ctDNA (personalized SNV-based)	Monitoring treatment response, early recurrence, and survival in USC and carcinosarcoma	Plasma	n = 16 (14 USCs and 2 CSs)	ddPCR based on tumor-informed SSVs via NGS (Foundation Medicine)	Bellone et al. (2023) [87]
Universally methylated ctDNA (*ZSCAN12*, *OXT*)	Highly specific and sensitive detection of EC; potential for diagnosis and monitoring	Plasma	Retrospective: 78 EC tumors + 30 adjacent; Prospective pilot: 33 EC (stage I–IV); Controls: 55 non-cancer individuals	Methylation-specific droplet digital PCR (meth-ddPCR)	Beinse et al. (2022) [68]
ctDNA as prognostic marker	Detection of ctDNA postoperatively significantly associated with progression and decreased OS	Plasma	n = 9	ddPCR	Feng et al. (2021) [90]
ctDNA (*PIK3CA* and *KRAS* mutations)	Presence of ctDNA in plasma correlated with advanced FIGO stage, non-endometrioid histology, LVSI, and poorer recurrence-free and overall survival	Plasma	n = 199 (68 had tumor mutations; 10 had matched ctDNA)	ddPCR (*PIK3CA*, *KRAS*)	Shintani et al. (2020) [85]
Uterine aspirate + cfDNA NGS	ctDNA present in 41.2% overall; enriched in high-risk subtypes	Uterine aspirates, plasma	n = 60	NGS panel	Casas-Arozamena et al. (2020) [55]
ctDNA mutation burden	Rising ctDNA precedes radiographic or clinical recurrence by months; captures emerging MSI; dynamic real-time monitoring	Serial plasma	n = 13	ddPCR + targeted NGS	Moss et al. (2020) [86]
cfDNA **content & integrity** index	Increased total cfDNA and Alu integrity ratio correlate with higher grade and LVSI, independent of hypertension or obesity	Serum	n = 60	qPCR–Alu quantification	Vizza et al. (2018) [91]
Serum HE4 and CA125	HE4 is more sensitive than CA-125 for detecting recurrence; elevated levels correlate with advanced stage, myometrial invasion, nodal metastases, and shorter survival	Serum	n = 174	Enzyme immunoassay	Abbink et al. (2018) [92]
Total cfDNA and cfmtDNA	Elevated total cfDNA in higher-grade tumors	Serum	n = 59 (12 G1, 30 G2, 17 G3)	RT-qPCR	Cicchillitti et al. (2017) [93]
CTC enumeration (CellSearch^®^)	CTC count correlated with stathmin expression and advanced disease	Whole blood	n = 30	CellSearch^®^ + immunofluorescence	Lemech et al. (2016) [94]
Personalized ctDNA panels	Personalized ctDNA detected residual disease and recurrence around 6 months ahead of CA-125 and imaging; associated with shorter PFS/OS	Tissue, serum	n = 44 (17 EC cases)	Tumor-specific ddPCR	Pereira et al. (2015) [11]
miR-135b, miR-205, miR-30a-3p	miR-135b and miR-205 elevated in tissue/plasma; levels drop post-hysterectomy	Tissue/plasma	n = 24	NGS + qRT-PCR	Tsukamoto et al. (2014) [95]
cfDNA, p53 autoantibody, KRAS mut	cfDNA/KRAS detected in 19% of Type II and 11.9% of higher-grade EC; autoantibodies in 20%	Plasma	n = 109 (87 Type I, 22 Type II)	PCR-RFLP	Dobrzycka et al. (2010) [96]

Abbreviations: AEH: atypical endometrial hyperplasia; AUC: area under the curve; CA-125: cancer antigen 125; cfDNA: cell-free DNA; cfmtDNA: circulating free mitochondrial DNA; CS: carcinosarcoma; CTCs: circulating tumor cells; ctDNA: circulating tumor DNA; ddPCR: droplet digital PCR; DNA: deoxyribonucleic acid; EC: endometrial cancer; FIGO: International Federation of Gynecology and Obstetrics; HE4: human epididymis protein 4; LVSI: lymphovascular space invasion; miRNAs: microRNAs; MSI: microsatellite instability; NCBI: National Center for Biotechnology Information; NGS: next-generation sequencing; OS: overall survival; PCR-RFLP: polymerase chain reaction–restriction fragment length polymorphism; PFS: progression-free survival; qPCR: quantitative polymerase chain reaction; qMSP: quantitative methylation-specific PCR; qRT-PCR: quantitative reverse transcription PCR; RT-qPCR: reverse transcription quantitative PCR; SSV: somatic structural variant; TCGA: The Cancer Genome Atlas; USC: uterine serous carcinoma; WID-qEC: Women’s cancer risk IDentification—quantitative polymerase chain reaction test for Endometrial Cancer.

**Table 2:** List of the recent clinical trials on liquid biopsies in endometrial cancer acquired from ClinicalTrials.gov.

Identifier	Title	Aim(s) and Intervention(s)	Study Type	Start Date (Actual)	Study Completion (Estimated)	Enrollment (Estimated)	Status
NCT05504161	Detection of Tumor DNA Through Cervical Smear and Liquid Biopsy in EC Patients and Evaluation of Prognostic and Predictive Values of Tumor DNA Assay	To compare the ctDNA mutation detection rate based on cervical swab and whole blood at the time of surgery	Observational	30 December 2020	December 2023	300	Unknown
NCT06846775	The Clinical Utility of DNA Methylation Testing in Patient-collected Urine and Vaginal Samples to Detect EC: a Case-control Study	Diagnostic Test: DNA-methylation testing of methylation markers *CDO1*, *GHSR* and *ZIC1* for patient-collected vaginal samples and *GHSR*, *CDH13* and *SST* for patient-collected urine samples	Observational [Patient Registry]	15 April 2025	30 November 2027	120	Recruiting
NCT04456972	Reliability and Interest of Circulating Tumor DNA in ECs	To determine the concordance rate between molecular analysis of tumor tissue and that of ctDNA in patients with EC during treatment	Interventional	19 June 2020	8 January 2022	44	Completed
NCT06341855	Exploring the Potential of ctDNA-MRD for Recurrence Surveillance and Prognostic Evaluation in High-risk EC	To explore the feasibility of ctDNA-MRD in monitoring recurrence and evaluating prognosis of high-risk endometrial carcinoma	Interventional	25 January 2024	30 January 2026	100	Recruiting
NCT05099978	Asian Multicenter Prospective Study of ctDNA Sequencing (A-TRAIN)	NGS analysis will be performed on cfDNA extracted from peripheral blood samples of target patients to determine the types and incidences of genetic abnormalities	Observational	1 November 2021	31 December 2024	506	Active, not recruiting
NCT03744962	MSI in Circulatory DNA of EC	to analyze the MSI in the circulatory tumor DNA and in the tumor tissue in the patients diagnosed with uterine EC	Observational	10 November 2018	23 December 2020	100	Unknown
NCT05366881	cfDNA Assay Prospective Observational Validation for Early Cancer Detection and Minimal Residual Disease (CAMPERR)	To train and validate a genome-wide methylome enrichment platform to detect multiple cancer types and to differentiate amongst cancer types, including EC	Observational	3 May 2022	December 2026	7000	Recruiting
NCT04651738	Cell-free DNA Methylation for EC	To perform methylation testing of host DNA, namely, *BHLHE22*, *CELF4*, *HAND2*, and *ZNF177*, in the peripheral serum to discover the diagnostic and supervision roles of DNA methylation in EC	Interventional	18 December 2020	1 January 2023	400	Unknown
NCT05955079	Circulating Tumor DNA Study in Patients With EC (ctDNA-endo)	To identify a population at risk of early recurrence after oncologic resection surgery of a primary uterine tumor based on the detection of ctDNA	Observational	1 January 2021	January 2026	130	Recruiting
NCT06083779	Early Detection of EC Using Plasma Cell-free DNA Fragmentomics	To enable non-invasive early detection of EC in high-risk populations through the establishment of a multimodal machine learning model using plasma cell-free DNA fragmentomics	Observational	1 August 2023	30 April 2024	216	Recruiting
NCT06028724	A Study on the Prevalence of Clinically Useful Mutations in Solid Tumor Characterized by NGS Methods on Liquid Biopsy Analysis (POPCORN) (POPCORN)	To evaluate the real-world prevalence of clinically useful mutations in patients who are receiving therapy for advanced and locally advanced solid tumor through liquid biopsy, including EC	Observational	26 May 2023	31 May 2030	782	Recruiting
NCT05059444	ORACLE: Observation of ResiduAl Cancer With Liquid Biopsy Evaluation (ORACLE)	To demonstrate the ability of a novel ctDNA assay developed by Guardant Health to detect recurrence in individuals treated for early-stage solid tumors, including EC	Prospective Cohort	7 September 2021	February 2028	1050	Recruiting
NCT05051722	Leveraging Methylated DNA Markers (MDMs) in the Detection of EC, Ovarian Cancer, and Cervical Cancer (ECHO)	To develop a pan-gynecologic cancer detection test using gynecologic (unique endometrial, cervical, and ovarian cancer) cancer-specific methylated DNA markers and high-risk human papilloma virus (HR-HPV) detected in vaginal fluid and/or plasma	Observational	3 August 2021	30 December 2026	3110	Recruiting
NCT05049538	Determine the Utility of Liquid Biopsies and Tumor Molecular Profiling in Predicting Recurrence in ECs	To find out how well liquid biopsies work as a non-invasive alternative to other methods of finding cancer cells (such as a tissue biopsy) in patients with EC by comparing *TP53*, *FBXW7* and other mutated genes in ctDNA samples obtained at several timepoints of disease progression/treatment	Observational	18 June 2019	30 June 2028	1000	Recruiting
NCT04817501	Phenotypic Spectrum of CTCs in Tumors of the Female Reproductive System	To evaluate the level and molecular profiles of different CTC populations as markers for predicting the risk of developing hematogenous metastases and the effectiveness of treatment in patients with tumors of the female reproductive system including EC	Observational	14 February 2014	1 December 2022	150	Completed [105]
NCT03776630	Exploring the Potential of Novel Biomarkers Based on Plasma miRNAs for a Better Management of Pelvic Gynecologic Tumors (GYNO-MIR)	To validate the 5-miR index assessed in plasma samples as a diagnostic marker to assess the risk of lymph node metastases	Interventional	23 May 2019	May 2027	363	Active, not recruiting

Abbreviations: cfDNA: cell-free DNA; CTCs: circulating tumor cells; ctDNA: circulating tumor DNA; DNA: deoxyribonucleic acid; EC: endometrial cancer; HR-HPV: high-risk human papillomavirus; miRNAs: microRNAs; MRD: minimal residual disease; MSI: microsatellite instability; NGS: next-generation sequencing; NCT: National Clinical Trial (identifier number from ClinicalTrials.gov).

## Data Availability

The original contributions presented in this review are included in the article. Further inquiries can be directed to the senior author.
